# Editing of a Novel Cd Uptake-Related Gene *CUP1* Contributes to Reducing Cd Accumulations in *Arabidopsis thaliana* and *Brassica napus*

**DOI:** 10.3390/cells11233888

**Published:** 2022-12-01

**Authors:** Junyu Yao, Jiuyuan Bai, Sha Liu, Jingyan Fu, Ying Zhang, Tianshun Luo, Hongpei Ren, Rui Wang, Yun Zhao

**Affiliations:** 1 Key Laboratory of Bio-Resource and Eco-Environment of Ministry of Education, College of Life Sciences, Sichuan University, Chengdu 610065, China; 2 Science and Technology Innovation Center of Sichuan Modern Seed Industry Group, Chengdu 611100, China

**Keywords:** Cd-safe crops, major facilitator superfamily, CRISPR/Cas9, agronomic traits

## Abstract

*Brassica napus* is a Cd hyperaccumulator, which is a serious threat to food and fodder safety. However, no related studies on developing Cd-safe *B. napus* have been reported yet. Here, we screened out a novel Cd uptake-related gene, *AtCUP1,* from the major facilitator superfamily in *Arabidopsis thaliana*. The mutation of *AtCUP1* decreased Cd accumulation, both in roots and shoots of *A. thaliana*. Furthermore, the disruption of the *AtCUP1* gene by the CRISPR/Cas9 system significantly reduced Cd accumulation in *A. thaliana*. Interestingly, the disruption of the *BnCUP1* gene, an orthologous gene of *AtCUP1*, by the CRISPR/Cas9 system also diminished Cd accumulation in both roots and shoots of *B. napus* based on the hydroponics assay. Furthermore, for the field experiment, the Cd accumulations of *BnCUP1*-edited lines were reduced by 52% in roots and 77% in shoots compared to that of wild-type (WT) lines, and the biomass and yield of *BnCUP1*-edited lines increased by 42% and 47% of that of WT, respectively. Noteworthily, agronomic characteristics of *B. napus* were not apparently affected by *BnCUP1*-editing. Thus, *BnCUP1*-edited lines are excellent non-transgenic germplasm resources for reducing Cd accumulation without a distinct compromise in yield, which could be applied to agricultural production in Cd-contaminated soils.

## 1. Introduction

Cadmium (Cd) is a heavy metal with high toxicity and persistent residue. Under the conditions of unreasonable industrial activities and wastewater irrigation, excessive Cd in agricultural soil has become more and more serious all over the world [1]. Due to high mobility in the soil-plant system, Cd is easily absorbed from soil by the root epidermal cell, and then translocated via the vascular system to various tissues of plants [2]. Oilseed rape, an important resource of edible vegetables, animal fodder and condiments, is one of the most widely produced oil crops in China and even plays a significant role in edible oil production worldwide [3]. However, oilseed rape has a high Cd accumulation capacity in stems and leaves [4], which not only causes an adverse effect on crop yields, but also further causes a great threat to human health through the food chain [5,6]. Therefore, it is quite essential to reduce Cd accumulation in the edible part of oilseed rape to ensure food safety for animals and humans. Current studies on reducing Cd accumulation in oilseed rape are mainly focused on the exogenous addition of chemical regulators in cropland soil [7,8,9,10]. However, these measures may introduce additional contaminants to the soil. Thus, the immediate challenge ahead is to seek a compelling alternative approach for creating low-Cd oilseed rape [11,12].

In general, the membrane-located metal transporter proteins, including nature resistance-associated macrophage proteins (NRAMP) and heavy metal P-type ATPases (HMA), are regarded as the most important channels responsible for Cd uptake, transfer and transport in plants [13,14,15,16]. Consequently, the genetic manipulation of these genes was gradually reported for cultivating low-Cd varieties of rice. However, many studies have found that disruption of these translocator proteins may cause adverse or unintended effects on plants. For example, editing Cd uptake-related genes such as *OsNRAMP1* and *OsNRAMP5* could actually reduce Cd content in rice, but simultaneously cause poorer quality and lower yields [11,12,17,18,19]. In addition, some studies found that the double mutants *athma2athma4* eliminated Cd translocation from root to shoot almost completely, but caused developmental retardation, yellowing and abortion [20,21]. While the genetically manipulated low-Cd rice varieties were well-documented, no example of low-Cd oilseed rape developed by molecular genetic tools has been reported as of yet. Given the adverse effect of the functional disruption of translocators on yields, together with cooperative interactions of multiple genes in crop Cd accumulation, it is, therefore, worthwhile to identify more gene resources involved in Cd accumulation for the purpose of cultivating Cd-safe and well-grown oilseed rape.

The major facilitator superfamily (MFS), as an ancient and ubiquitous transporter family, is one of the largest secondary active transporter families, and is mainly responsible for secondary metabolites transportation in eukaryotes [22]. Two members of the MFS superfamily have been found to be involved in Cd uptake [11,23]. Therefore, it is possible to discover more genes associated with cadmium absorption in this family.

Here, we screened out and identified a Cd uptake-related gene, *AtCUP1,* from the *A. thaliana* MFS family. The disruption of the *AtCUP1* gene by the CRISPR/Cas9 system reduced Cd accumulation in *A. thaliana* and had no adverse effect on the plant growth. Since *A. thaliana* is closely related to *B. napus*, we disrupted the homologous gene *BnCUP1* in *B. napus* by the CRISPR/Cas9 system. The *BnCUP1*-edited lines growing in cadmium-contaminated fields displayed a significantly higher biomass, seed yield and a sharply lower Cd accumulation than WT. Furthermore, no apparently deleterious effects on agronomic characteristics were observed in *BnCUP1*-edited lines in non-Cd-contaminated fields, which provided a compelling low-Cd oilseed rape germplasm for practical applications.

## 2. Materials and Methods

### 2.1. Phylogenetic Analysis of AtCUP1

The phylogeny of 128 *A. thaliana* MFS proteins obtained from Transport Database (http://www.membranetransport.org/transportDB2/index.html, accessed on 1 November 2019) was constructed by using the neighbor-joining method in MEGAX [15]. EvolView (http://evolgenius.info/#/, accessed on 2 November 2019) was used to visualize the phylogenetic tree [24]. The gene symbol IDs of 128 *A. thaliana* MFS genes, including these unclassified genes, are shown in Supplementary Figure S1.

### 2.2. Cadmium Transport Activities Assay in Yeast

The entire coding sequence (CDS) of these unclassified genes (A-I) was amplified from the WT *A. thaliana* cDNA by the specific primers (Supplementary Table S1), and then inserted into pYES2 plasmid backbone using restriction cloning so that the CDS was under the control of *GAL1* promoter. The orientation and sequence of inserted CDS were confirmed by Sanger sequencing (Tsingke Biotechnology, Chengdu, CHN). The competent cells of the Cd-sensitive yeast expression system *Δycf1* were prepared based on a previous report [25]. Briefly, the activated yeast cells were inoculated into YPDA medium at a ratio of 1:100 for further growth to OD_600_ at 0.4~0.6. The cells were pelleted by centrifugation at 3000× *g* for 5 min at room temperature, and were then resuspended by 500 μL TE/LiAc (containing 50 μL 10 × TE buffer, 50 μL 1 M LiAc, 400 μL ddH_2_O) after washing once with sterile water. Next, 2 μg plasmid, 500 μL PEG/LiAc (including 400 μL 50% PEG, 50 μL 10 × TE and 50 μL 1M LiAc) and 60 μL yeast competent cells were mixed and incubated at 30 °C for 30 min, followed by an incubation with 30 μL DMSO for 15 min at 42 °C. The mixture was centrifuged at 8000× *g* and resuspended by 500 μL 1 × TE buffer. The yeast transformants were spread on SD-Ura solid medium (with 2% glucose) and incubated at 30 °C for 48 h.

For the plate growth test, yeast transformants were cultured to OD_600_ at 1.0. The culture was subsequently diluted to OD_600_ at 0.1, 0.01, 0.001 and 0.0001 by the sterile water. Then, the yeast transgenic strains were spotted on SD-Ura solid medium (with 2% galactose) with and without 30 μM Cd, respectively. Yeast-carrying empty vectors were used as controls. Yeast transformants were incubated at 30 °C for 4–5 d for the phenotype observation. All tests were performed in triplicate, and the experiments repeated independently three times.

### 2.3. Subcellular Localization Assay

Protoplasts were prepared based on a previous report [26], with some modification. In brief, the 4-week-old *Nicotiana benthamiana* leaves were cut into strips and placed in enzymatic hydrolysis solution containing 1.5% Cellulase R10, 0.4% Macerozyme R10, 0.4 M mannitol, 20 mM KCl and 20 mM MES at 28 °C for 3 h in darkness in order to obtain protoplasts. The CDS of *AtCUP1* was inserted into the pBI221 plasmid backbone between two cleavage sites of the restriction enzyme (*Xba*I and *Bam*HI) to obtain CaMV*35S*::*AtCUP1-GFP* recombinant plasmid. The mixture of 1 μg pBI221-AtCUP1-GFP recombinant plasmid and 230 μL 40% PEG solution, containing 0.8 g PEG, 0.2 M mannitol and 100 mM Ca (NO_3_)_2_, were added into 200 μL protoplast solution and gently mixed for the transformation at room temperature for 15–20 min. The transformation was stopped by adding 4 times volume of W5 solution, containing 154 mM NaCl, 125 mM CaCl_2_, 2 mM MES and 5 mM KCl. The mixture was centrifuged at room temperature 100× *g* for 2 min, and the protoplasts were washed by 1 mL W5 solution. Then, it was centrifuged again and resuspended by 1 mL WI containing 0.5 M mannitol, 20 mM KCl and 4 mM MES. The transformed protoplasts were cultured in darkness at 25 ℃ for 6–8 h to express fusion proteins. The protoplast images were captured by a fluorescence confocal microscope (Leica TCS SP5 II).

### 2.4. Analysis of AtCUP1 Promoter Activity

The 800 bp region upstream *AtCUP1* was amplified from the genome DNA of WT *A. thaliana* by specific primers (Supplementary Table S1) and then cloned into pDX2181 plasmid backbone between two cleavage sites of the restriction enzyme (*Hind*III and *Bam*HI) to obtain *proAtCUP1::GUS* recombinant plasmid. The *proAtCUP1::GUS* construct was transformed into *Agrobacterium* GV3101 competent cells and subsequently introduced into *A. thaliana* by *Agrobacterium tumefaciens*-mediated inflorescence infection. The T_0_ generation seeds which we obtained were screened on the solid MS medium containing 25 μg/mL Hygromycin. Two-week-old Hygromycin-resistant seedlings were transferred to soil for 10 days of growth, followed by a positive transgene identification using genome PCR with the primer pairs Pro-4G-F/R (Supplementary Table S1). Then, homozygous transgene lines with single insertion loci were selected by segregation ratio of Hygromycin resistance and used in subsequent experiments. The 10-day-old homozygous transgenic seedlings were transferred on 1/2 MS solid medium with and without 10 µM Cd for another 5 days of growth. Histochemical analysis of GUS activity was performed. In order to determine GUS activity, the samples were incubated into GUS dye solution (Solarbio, G3061, Beijing, CHN) at 37 °C for 24 h.

After being stained, chlorophyll was removed with 75% ethanol. The staining sites of the plants were imaged under a microscope.

### 2.5. Preparation of the AtCUP1-Overexpressing Transgenic Lines, atcup1 Mutant and CRISPR-AtCUP1 Lines

The CDS of *AtCUP1* was amplified by using the specific primers (Supplementary Table S1), and then cloned into the vector pBI121 plasmid backbone between two cleavage sites of the restriction enzyme (*Xba*I and *Xma*I) to construct the pBI121-*AtCUP1* plasmid. Then, the plasmid pBI121-*AtCUP1* was transformed into *Agrobacterium* GV3101. The WT *A. thaliana* (Col-0) was used as a host to obtain *AtCUP1-*overxpressing transgenic line (*OE*) by *Agrobacterium tumefaciens*-mediated inflorescence infection [27]. Three homozygous overexpressing lines (*OE#5-2, OE#6-1, OE#8-1*) were selected, based on the relatively high expression levels of *AtCUP1,* for Cd stress studies.

*Atcup1* mutant seeds were purchased from the Arabidopsis Biological Resource Center (ABRC, https://abrc.osu.edu/, accessed on 1 January 2020). Homozygous mutants were identified by the three-primer method, for which we referred to the website (http://signal.salk.edu/tdnaprimers.2.html, accessed on 1 June 2020). The primers used for identification are listed in Supplementary Table S1.

*CRISPR-AtCUP1* lines (*CR-1, CR-2, CR-3*) were obtained. First, the targets were designed using the CRISPR-P2.0 tool (http://crispr.hzau.edu.cn/CRISPR2/, accessed on 20 July 2020). Then, the CRISPR/Cas9 plasmid was constructed according to the description by Wang and Chen [28]. In brief, the targets were cloned into sgRNA-expressing vector pAtU6-26-M to form a pAtU6-26-sgRNA construct. The pAtU6-26-sgRNA constructs were subsequently fused into Cas9-expressing vector pUBQ10:Cas9-P2A-GFP (a kind gift from Haodong Chen) to form pAtU6-26-sgRNA- pUBQ10-Cas9-P2A-GFP plasmids by In-Fusion cloning. The constructed vector was transformed into *Agrobacterium tumefaciens* GV3101 competent cells. *A. thaliana* (Col-0) was used as a host to obtain CRISPR/Cas9*-AtCUP1* transgenic plants by *Agrobacterium tumefaciens*-mediated inflorescence infection. Total DNA was extracted from CRISPR/Cas9*-AtCUP1* positive transformation lines. Finally, the genomic region around the sgRNA target was amplified by PCR using specific primers (Supplementary Table S1), and the amplicons were directly identified by Sanger sequencing to identify mutations.

### 2.6. Cd Treatment Experiment of Different Growth Stages

In order to investigate the response of *OE*, *atcup1* and WT *A. thaliana* to Cd stress at different growth stages, all plants germinated on the 1/2 MS nutrient medium for 3 days. The plants with consistent growth were selected and transferred to the 1/2 MS nutrient medium, with and without 60 µM Cd, for another 7 days of growth. The root length was measured by ImageJ software. For hydroponic experiments, *A. thaliana* seeds of *OE*, *atcup1* and WT lines were sown in the 1/2 MS nutrient medium, and the five-day-old plants were transferred to 1/4 × Hoagland solution for 5 days’ growth. After that, the seedings were transferred to 1/2 × Hoagland solution for 4 days’ growth. In the end, the seedings were transferred to 1 × Hoagland solution, with and without 2.5 µM Cd, for 7 days’ treatment.

In the pot experiment, one-week-old seedlings of *OE*, *atcup1* and WT *A. thaliana,* growing in the 1/2 MS nutrient medium, were transplanted to pots with 2.13 mg/kg Cd until ripening for sampling. The plants growing in the pot soil without an additional Cd supplement were grouped as controls. The dry weight and the Cd accumulation were measured using the previous method [29].

### 2.7. Localization Experiment of Cd in Tissues

One-week-old seedlings of *OE*, *atcup1* and WT *A. thaliana,* growing in 1/2 MS solid medium with and without 60 µM Cd, were sampled and washed with deionized water. The washed seedlings were exposed to the staining solution (30 mg dithizone, 60 mL acetone, 20 mL water and 100 µL glacial acetic acid) for 1 h. Afterward, they were briefly washed in deionized water, for which we referred to the experiment described in [30]. The cadmium–dithizone precipitate was a reddish-brown precipitate.

### 2.8. Quantitative Real-Time PCR

The wild-type *A. thaliana* and *B. napus* were treated with 50 μM Cd for 48 h. The total RNA was extracted from roots, stems, leaves, silique and seeds to be reversely transcribed into cDNA as qPCR template. The double distilled water (ddH_2_O) was used as a negative control template. The qPCR was performed by using SYBR Green PCR Master Mix (Vazyme, Q712-02, Nanjing, CHN). The *AtActin* 7 gene was used for the internal standard, and the 2^−ΔΔCt^ method was used for calculating the relative expression level [31]. All primers are shown in Supplementary Table S1.

### 2.9. Construction and Identification of BnCUP1-Edited Lines

The sequence of *BnCUP1* was found by BLASTp analysis in the NCBI database (https://www.ncbi.nlm.nih.gov/, accessed on 20 October 2020). The target sequence was designed by using CRISPR-P2.0 (http://crispr.hzau.edu.cn/CRISPR2/, accessed on 22 October 2020) website. The selection basis of the target was that the sgRNA targeted *BnCUP1-1b* and *BnCUP1-1c*. The PKSE401 plasmid was used to construct the CRISPR/Cas9-*BnCUP1* recombinant plasmid, and the vector construction process was performed by referring to Xing et al. [32]. The *B. napus* (Westar) was used as the host to obtain *CRISPR/Cas9-BnCUP1* transgenic plants by means of *Agrobacterium tumefaciens*-mediated infection [33]. The DNA of T_0_ generation *B. napus* was extracted by a DNA extraction kit (Transgen, EE111-01, Beijing, CHN), and positive transgenic lines were identified by PCR using the extracted DNA as a template. Finally, 50 positive transgenic plants were obtained.

For identification of *BnCUP1*-edited lines, the genomic region around the target of the *BnCUP1* gene was amplified from the 50 positive transgenic *B. napus* by using specific primers (Supplementary Table S1), and the PCR fragments were amplified by secondary PCR to add sequencing connectors. The products were sequenced by using paired-end 150 bp sequencing (PE150) [34]. For off-target analysis, specific primers (Supplementary Table S1) around the putative off-target sites were used for PCR amplification, and the products were sequenced by using Sanger sequencing. The process of detection of potential off-target sequences is shown in Supplementary Table S2. Finally, we obtained eight *BnCUP1*-edited lines, two of which were doubly edited at both *BnCUP1-1b* and *BnCUP1-1c* gene loci, termed as *S12* and *S34.*

### 2.10. Hydroponic Experiments of BnCUP1-Edited Lines

Seeds of *BnCUP1*-edited lines (*S12* and *S34* lines) germinated in wet filter paper, and five-day-old seedlings were transferred to 1/4 × Hoagland solution to grow for 4 days. After that, the *BnCUP1*-edited lines grew in 1/2 × Hoagland solution for 4 days. Then, they grew in 1 × Hoagland solution for 2 days. In the end, the *BnCUP1-*edited lines grew in 1 × Hoagland solution with and without 4 µM Cd for 20 days. Westar was used as a control. The plants were cultured in a growth chamber at 23 °C, and the nutrient solution was changed every 2 days. The aboveground and underground parts were collected to determine the biomass and Cd concentration.

### 2.11. The Field Experiment for CRISPR-BnCUP1 Lines

The field experiment was carried out by planting cas9-free *BnCUP1*-edited lines in contaminated soil near a factory of Chengdu Plain, from October 2021 to May 2022. The field soil with low-Cd concentration was used as a control. A random complete block design was used in the field experiment. The inter-row spacing of all seedlings was 18 cm, and the space between columns was 20 cm. At least 30 plants were planted for each type of line. Agronomic traits, biomass and Cd accumulation were measured at harvest.

### 2.12. Determination of Metal Elements

The relative contents of Cd, Zn, Mn and Fe in plant samples were determined by an Inductively Coupled Plasma Emission Spectrometer (Thermo Fisher Scientific, MA, USA, ICAP-7200). The 0.2 g dried sample was placed into a conical bottle to add 5 mL HNO_3_ and 1 mL HClO_4_. The solution was digested by a graphite digesting instrument at 200°C until the solution became colorless and transparent in the conical bottle. After cooling, the digested solution was poured into a 50 mL volumetric flask, and was calibrated to 50 mL with distilled water. All digestion took place in three replicates. All tests were performed at least three times, with blank HNO_3_ as negative control.

## 3. Results

### 3.1. Screening of Cd Uptake-Related Genes

**Figure 1:**
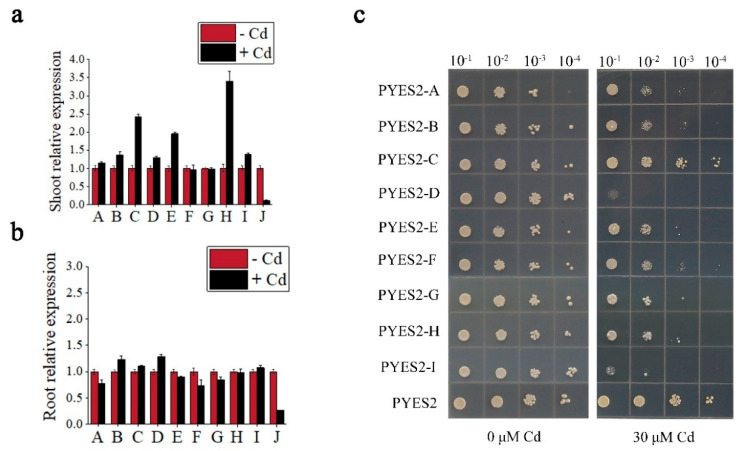
Screening of Cd uptake-related candidate genes. (**a**,**b**) The expression level of candidate genes in *A. thaliana* with or without 50 μM Cd treatment. (**c**) Dilution-series spot assays of yeast strains treated with or without 30 μM Cd. Error bars indicate standard deviation of means. Experiments in (**a**–**c**) were independently repeated three times with similar results.

The *A. thaliana* MFS superfamily contains 128 members, of which 118 have been reported to transport various substrates, and the remaining 10 proteins (A-J) have not been studied on their transport substrates. Phylogenetic analysis of 128 MFS members showed that the proteins facilitating the same substrate transport shared a closer phylogenetic relationship, while 10 proteins separately formed four unknown-function clades (Supplementary Figure S1). In order to investigate the expression profile of the 10 unknown genes’ responses to Cd stress, the expression level of these genes in root and shoot were analyzed by RT-qPCR. The results showed that the transcripts of all genes were detected in roots and shoots without Cd treatment, while only the B, C, D and I genes showed significantly up-regulated expression in both roots and shoots after Cd stress (Figure 1a,b).

In order to evaluate whether these genes are associated to Cd uptake, spot assays were carried out on plates, with or without 30 µM Cd. The growth rate was normalized by yeast strains transformed with empty vectors. All transgenic strains showed no differential growth with controls on Cd-free plates (Figure 1c), while yeast transformed with pYES2-D displayed a visibly distinct Cd sensitivity compared to the other yeast transgenic strains after 30 µM Cd exposure. These results indicated that the gene D was the most likely candidate involved in Cd uptake, which was christened *AtCUP1* (*A. thaliana* cadmium uptake-related protein).

### 3.2. Expression Pattern Analysis of AtCUP1

**Figure 2:**
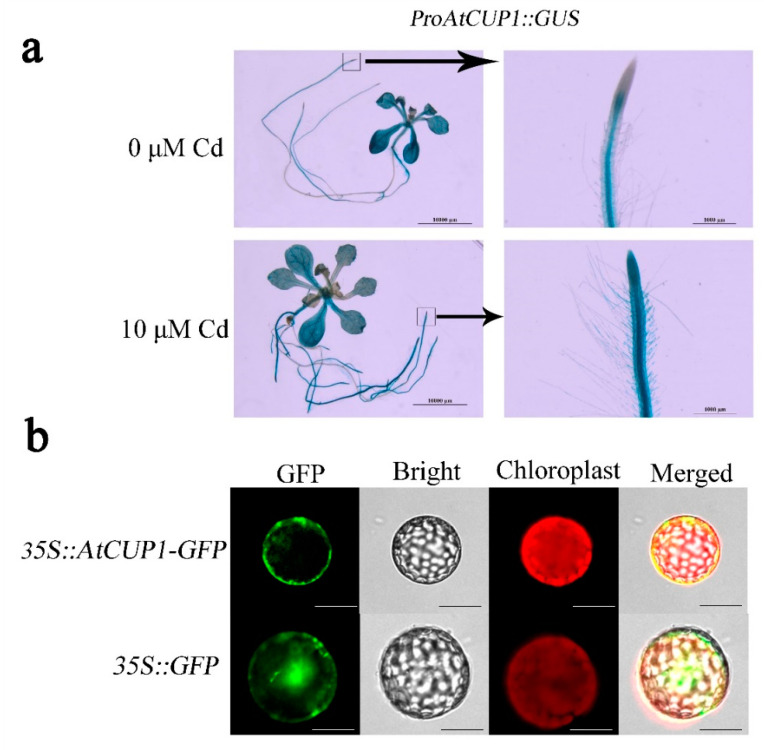
Expression pattern analysis of *AtCUP1* in *A. thaliana*. (**a**) Tissue-dependent expression of *AtCUP1* in *A. thaliana* detected by GUS histochemical assay of the transgenic plant with the GUS reporter driven by the *AtCUP1* promoter. The right panels are zooms of the boxed area in the left panel. Bars indicate 10 mm in the left panels and 1 mm in the right. (**b**) Subcellular location of AtCUP1 in *N*. *benthamiana* protoplasts by fusion GFP. Bars indicates 20 μm.

In order to determine the tissue-dependent expression of *AtCUP1*, GUS histochemical analysis of transgenic *A. thaliana,* with the *GUS* reporter gene driven by the *AtCUP1* promoter, was performed. The results showed that the promoter activity of *AtCUP1* was detected in both roots and shoots under Cd-free conditions. After 10 µM Cd treatment, the promoter activity of *AtCUP1* was apparently increased in the root tip and lateral root compared to that under control conditions, while no significant difference was observed in the leaves (Figure 2a), which indicates that expression of *AtCUP1* in roots is induced by Cd stress. In order to further examine the subcellular localization of AtCUP1 protein, the fluorescence of the *N*. *benthamiana* protoplast expressing AtCUP1-GFP fusion protein was captured. The diffused fluorescence signal was detected in both the cytoplasm and the nucleus of the protoplast expressing GFP alone, while a distinct plasma membrane-located fluorescence signal of the AtCUP1-GFP fusion protein was observed (Figure 2b), which was consistent with the results predicted by bioinformatics (Supplementary Figure S2). Together with spot assay, it was suggested that the plasma membrane protein AtCUP1, which has inducible expression by Cd in roots, was likely involved in Cd uptake of *A. thaliana*.

### 3.3. AtCUP1 Was Confirmed to Be Involved in Cd Uptake in A. thaliana

**Figure 3:**
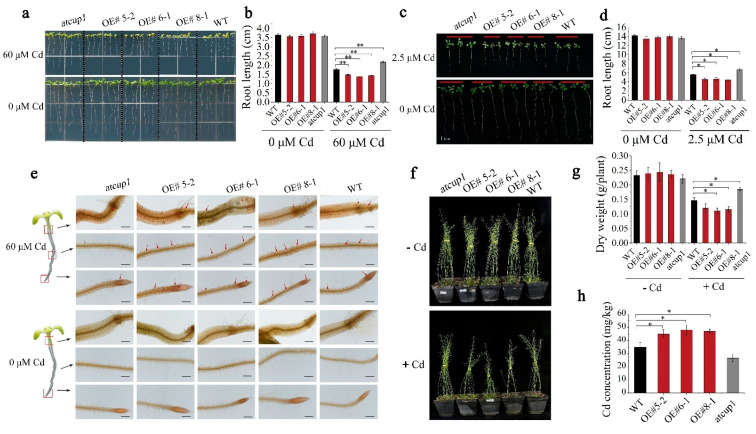
*AtCUP1* contributes to Cd absorption and accumulation in *A. thaliana*. (**a**) Growth status of three *A. thaliana* lines growing on 1/2 MS medium, containing 0 or 60 μM Cd. (**b**) Root length measurements of three *A. thaliana* lines in (**a**). growth status (**c**) and root length measurements (**d**) of *A. thaliana* cultured in hydroponic solution containing 0 or 2.5 μM Cd. (**e**) Visualization of Cd accumulation in the *A. thaliana* hypocotyl exposed to 0 or 60 μM Cd. The red arrows point to the precipitates of Cd-dithizone. Bars indicate 500 μm. (**f**) Growth status of three *A. thaliana* lines at harvest, growing in Cd-contaminated pot soil. (**g**,**h**) Dry weight and Cd concentration of three *A. thaliana* lines with and without Cd treatment. All data were obtained from three independent triplicate experiments and the error bar represents standard deviation. Statistical analysis was performed by Student’s *t-*test (* *p* < 0.05, ** *p* < 0.01).

In order to further investigate whether *AtCUP1* was involved in Cd uptake in *A. thaliana*, the *OE* lines and *atcup1* mutant were constructed for Cd stress treatment. In the seedling stage, the *OE*, *atcup1* and WT showed no phenotypic difference in Cd-free MS medium, while the *atcup1* mutant displayed better growth (Figure 3a) and longer roots than *OE* and WT under 60 µM Cd (Figure 3b). In vegetative stage, 2-week-old seedlings were cultivated in liquid nutrient solution with or without 2.5 µM Cd (Figure 3c). The results showed that the *atcup1* mutant lines displayed better growth and longer roots than WT and *OE*, which was in accordance with the results of the seedling stage assay (Figure 3d). In order to further depict that differential growth appeared to be a consequence of Cd accumulation, a dithizone staining experiment was employed to visualize Cd content in roots. The results showed that much lower quantities of reddish-brown precipitates of cadmium–dithizone were observed in the *atcup1* mutant compared with the other two lines (Figure 3e). These results indicate that the disruption of *AtCUP1* contributes to alleviating *A. thaliana* growth inhibition by decreasing Cd uptake. Under the control case, the growth was not affected by the mutation of *AtCUP1*. However, the *atcup1* mutant showed better growth than *OE* and WT after suffering from Cd stress (Figure 3f). Moreover, the *OE*, *atcup1* and WT showed a parallel dry weight under no Cd treatment. After Cd stress, the dry weight of *OE* was significantly lower than that of WT, while the *atcup1* mutant possessed a significantly higher dry weight than WT (Figure 3g). As for Cd accumulation, the results showed that the functional loss of *AtCUP1* decreased *A. thaliana* Cd concentration, while over-functional *AtCUP1* increased Cd concentration (Figure 3h), which was in agreement with the dithizone staining results. Together with the experiment results from the seedling stage and vegetative stage, it is suggested that *AtCUP1* is involved in Cd uptake and accumulation in *A. thaliana*.

### 3.4. Various AtCUP1-Edited Lines Showed Divergent Cd Accumulation

**Figure 4:**
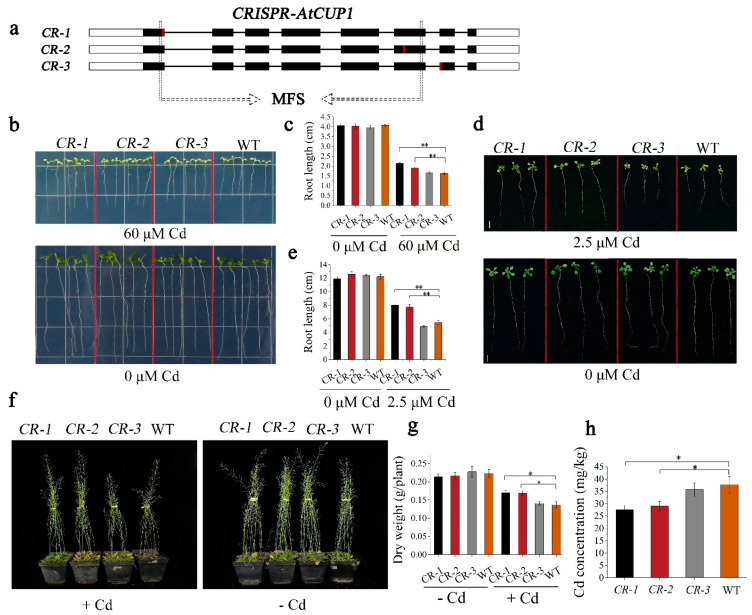
Disruption of the AtCUP1 domain enhanced Cd tolerance and reduced Cd accumulation. (**a**) Schematic diagram of three *CRISPR-AtCUP1* lines. The rectangle inside the dotted line indicates the MFS domain. The black rectangle marks the exon of *AtCUP1*, the straight line shows the intron of *AtCUP1*, and the red box indicates the location of the sgRNA. (**b,c**) Growth and root length measurements of three *CRISPR-AtCUP1* lines growing in 1/2 MS medium containing 0 or 60 μM Cd. (**d,e**) Growth and root length measurements of three *CRISPR-AtCUP1* lines cultured in hydroponic solution containing 0 or 2.5 μM Cd. (**f**) Growth status of three *CRISPR-AtCUP1* lines grown in pot soil with or without Cd. (**g**,**h**) Dry weight and Cd concentration of three *CRISPR-AtCUP1* lines grown in pot soil with and without Cd. Three independent experiments, each in triplicate, were performed for each measurement. The error bar represents standard deviation. Statistical analysis was performed by Student’s *t-*test (* *p* < 0.05, ** *p* < 0.01).

The CRISPR/Cas9 system was employed to further examine the role of *AtCUP1* in Cd uptake and accumulation. The sgRNAs against three different sites in and out of MFS domain of AtCUP1 were designed (Figure 4a). We have selected three types of homozygous *AtCUP1*-edited lines (*CR-1*, *CR-2* and *CR-3*) for the Cd treatment experiment, which consisted of an insertion of one base at the different target sites (Supplementary Figure S3). In the seedling stage, all of the seedlings growing in Cd-free conditions showed a similar growth status, while after Cd exposure, *AtCUP1*-edited lines showed apparently better growth than WT (Figure 4b). Moreover, the roots of *CR-1* and *CR-2* were longer than that of WT, while no significant difference between *CR-3* and WT was found (Figure 4c). In the hydroponic experiment, better growth (Figure 4d) and longer roots (Figure 4e) of *CR-1* and *CR-2* were observed compared with *CR-3* and WT after Cd stress, while no difference was observed without Cd stress. Furthermore, the Dithizone staining granted direct visual access to Cd content in roots. The results showed that there was decreased Cd accumulation in the roots of *CR-1* and *CR-2* compared with *CR-3* and WT (Supplementary Figure S4), which could account for different growth rates of *A. thaliana* seedlings. In the pot assay, all lines grew in Cd-containing soil until maturity. The growth was not affected by the functional disruption of *AtCUP1* in the control case, while a better growth of *CR-1* and *CR-2* was observed compared with WT and *CR-3* (Figure 4f). Both *CR-1* and *CR-2* showed increased dry weight compared to CR-3 and WT (Figure 4g). The Cd contents of *CR-1* and *CR-2* decreased by 27% and 23% more than those of WT, respectively, while the Cd content of *CR-3* was not significantly different from that of WT (Figure 4h). In conclusion, the mutation in different regions of *AtCUP1* caused a divergent ability of Cd uptake in *A. thaliana*. The CRISPR/Cas9-mediated gene editing against the first and sixth exon of *AtCUP1* significant increased *A. thaliana* Cd tolerance and decreased Cd accumulation, which provides a compelling Cd-uptake gene resource and paves the way for the further development of low-Cd-accumulation oilseed rape by gene editing.

### 3.5. CRISPR-BnCUP1 Mutation Reduced Cd Accumulation in B. napus

**Figure 5:**
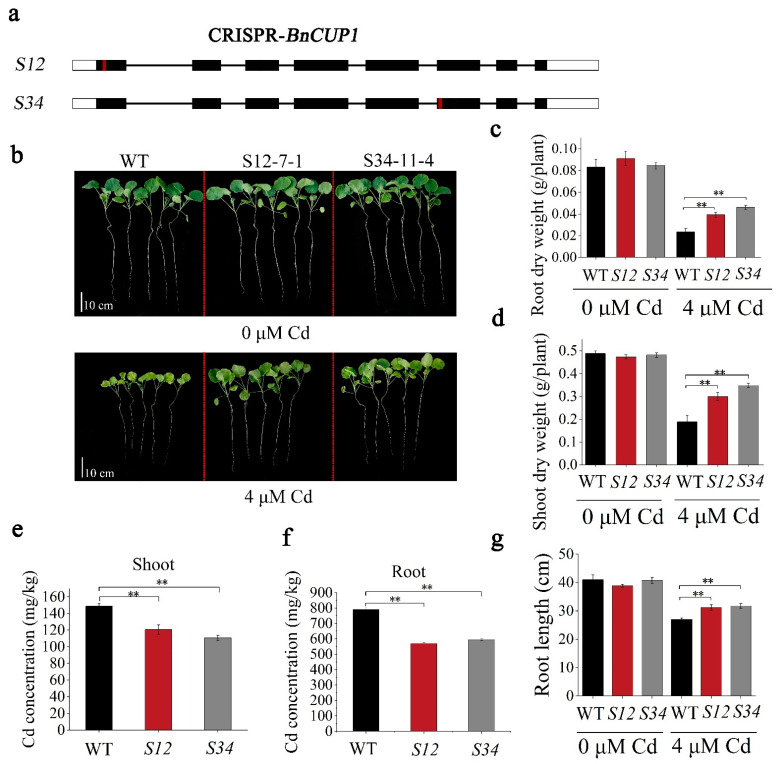
*BnCUP1*-edited *B. napus* reduced Cd accumulation. (**a**) Schematic diagram of two *CRISPR-BnCUP1* lines. The black rectangle shows the exon of *BnCUP1*, the straight line marks the intron of *BnCUP1*, and the red box indicates the location of the sgRNA. (**b**) Growth of two *CRISPR-BnCUP1* lines (*S12* and *S34*) cultured in hydroponic solution containing 0 or 4 μM Cd for 20 days. (**c**,**d**) Dry weight of WT, *S12* lines and *S34* lines in roots and shoots, respectively. (**e**,**f**) Cd concentration of WT, *S12* lines and *S34* lines in roots and shoots, respectively. (**g**) Root length of WT, *S12* lines and *S34* lines, respectively. All experiments were independently repeated at least three times. The error bar represents standard deviation. Statistical analysis was performed by Student’s *t-*test (** *p* < 0.01).

There were four homologous *CUP1* genes (LOC106376121, LOC106376123, LOC106403144, LOC106378223) in *B. napus,* named *BnCUP1-1a*, *BnCUP1-1b*, *BnCUP1-1c* and *BnCUP1-1d*. The similarity of the four genes was as high as 95% in amino acid levels (Supplementary Figure S5). The expression profile results showed that both *BnCUP1-1b* and *BnCUP1-1c* showed an up-regulated expression after Cd treatment (Supplementary Figure S6a), and a constitutive expression in various tissues of *B. napus* at different stages (Supplementary Figure S6b,c). Considering the possible role of *BnCUP1-1b* and *BnCUP1-1c* in Cd uptake, we have selected two types of *CRISPR-BnCUP1* lines, in which *BnCUP1-1b* and *BnCUP1-1c* were simultaneously edited in the first or sixth exon region, respectively (Figure 5a, Supplementary Figure S7 and Supplementary Table S2).

In order to test whether the *CRISPR-BnCUP1* lines reduced Cd uptake, 2-week-old seedlings (*S12*/*S34*/WT) were cultured with nutrient solution, with or without 4 µM Cd, for 20 days, and the Westar cultivar was used as a control (Figure 5b). Under Cd treatment, the growth status of the *CRISPR-BnCUP1* lines was superior to that of WT. The root length and dry weight of shoots and roots of *S12* increased by 16%, 59% and 66% of that of WT, respectively. The Cd contents in the shoots and roots of *S12* decreased by 19% and 28% of that of WT, respectively. The root length and the dry weight of shoots and roots of *S34* increased by 18%, 85% and 95% than that of WT, respectively. The Cd contents in the shoots and roots of *S34* declined by 32% and 25%, respectively, compared to those of WT (Figure 5c–g). The results of the control treatment showed that both the *CRISPR-BnCUP1* (*S12* and *S34*) and WT grew well in nutrient solution without Cd. In summary, these results indicate that the *CRISPR-BnCUP1* lines reduced Cd accumulation in *B. napus*.

### 3.6. BnCUP1-Edited B. napus Was Identified as a Compelling Low-Cd Germplasm

**Figure 6:**
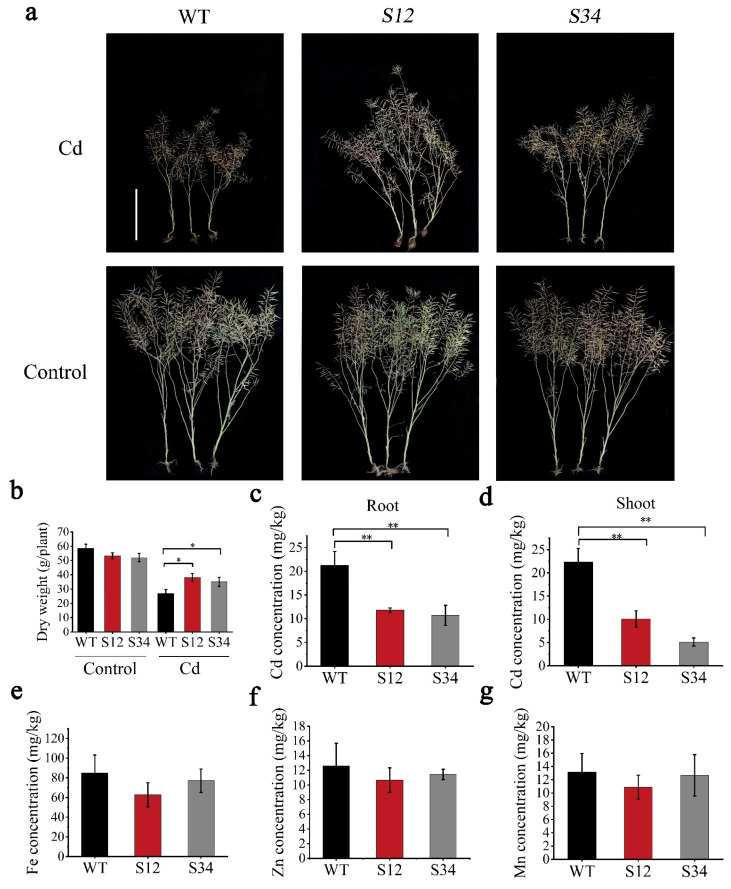
The field experiment for *CRISPR-BnCUP1* lines. (**a**) Growth status of three lines (WT, *S12* and *S34*) planted in Cd-contaminated fields and control fields. Bars indicate 50 cm. (**b**) Dry weight of seven-month-old WT, *S12* and *S34* oilseed rape grown in Cd-contaminated fields and control fields. (**c**,**d**) Cd concentration in roots and shoots of WT, *S12* and *S34* oilseed rape, respectively. (**e**,**f**,**g**) Concentrations of Fe, Zn and Mn in WT, *S12* and *S34* oilseed rape grown in Cd-contaminated fields. All data were obtained from independent triplicate experiments and the error bar represents standard deviation. Statistical analysis was performed by Student’s *t-*test (* *p* < 0.05, ** *p* < 0.01).

In order to assess the effect of editing *BnCUP1* on the plants’ growth, the field trial was performed by planting the *BnCUP1-*edited oilseed rapes in field with 5.12 mg/kg Cd. Plants grown in fields with 0.14 mg/kg Cd served as controls. The soil’s physicochemical properties are presented in Supplementary Table S3. At harvest, the growth status (Figure 6a), dry weight (Figure 6b) and agronomic characters (Supplementary Table S4) of *BnCUP1*-edited lines in control soil were not affected compared with WT. When under Cd stress conditions, *BnCUP1*-edited lines displayed superior growth to WT (Figure 6a), and the dry weight of *S12* and *S34* increased by 47% and 40% of that of WT, respectively (Figure 6b). Statistical results of agronomic traits showed that the yield of the *S12* line and the *S34* line increased by 41% and 47% of that of WT, respectively, and except for the 1000-seed weight, other agronomic traits of *BnCUP1*-edited lines, such as the number of seeds per silique, the plant height and the silique number per plant were superior to those of WT (Table 1). Furthermore, the Cd concentration in the shoots of the *S12* and *S34* lines decreased by 55% and 77% of that of WT, respectively. The Cd concentration in the roots of the *S12* and *S34* lines showed a 44% and 52% decrease compared to that of WT, respectively (Figure 6c,d). Moreover, the concentrations of trace elements (Fe, Zn and Mn) in the *S12* line exhibited no significant difference from that of WT, and this was the same case in the *S34* line (Figure 6e–g). In summary, compared with WT, the *CRISPR-BnCUP1* line reduced Cd accumulations and kept higher yields in Cd-contaminated soil, and is an excellent germplasm resource.

## 4. Discussion

Oilseed rape, apart from acting mainly as an edible vegetable oil, is also an important green vegetable favored by many people [35]. Nowadays, it is highly valuable to create new germplasm resources from vegetables, and oilseed rape could be used to satisfy the needs of the continuously altering diet structure. However, many studies have shown that the oilseed rape has a high ability to uptake Cd from Cd-contaminated soil [36,37,38], which causes a threat to human health. Consequently, the development of low-Cd-uptake oilseed rape varieties is essential to ensuring food safety.

In this study, based on the yeast spot assay, which is a widely used tool to screen Cd-uptake related gene [39], we screened out a novel Cd-uptake related gene from the unknown transporters-coding genes belonging to the MFS family in *A. thaliana*. The mutation of *AtCUP1,* caused by either T-DNA insertion or CRISPR/Cas9 mediated gene editing, apparently decreased Cd accumulations in both roots and shoots of *A. thaliana*, which, together with the cytomembrane location and high expression in roots, indicates that AtCUP1 is mainly responsible for Cd uptake. Given that different degrees of disruption in the AtCUP1 protein may cause a divergent ability of Cd uptake and an adverse effect on the growth of plants [17,18,19], we designed three editing sites against the *AtCUP1* gene. The result showed the edited lines. With the mutation occurring in the first and sixth exon of *AtCUP1,* it showed better growth and a lower level of Cd concentration than WT, while the seventh exon edited lines showed no difference in growth or Cd concentrations compared to WT (Figure 4). These results indicate that from the first to the sixth exon is the main domain responsible for Cd uptake, which is in agreement with the predicted MFS domain of AtCUP1 (Supplementary Figure S2). The recent study on the *OsCd1* gene, a member of the MFS superfamily protein, showed a similar result [11]. More interestingly, previous studies showed that the mutation of some membrane transporter-coding genes, such as *OsNramp1*, *OsNramp5*, *OsHMA3* and *OsHMA2*, caused different degrees of adverse effect on growth and yield under control conditions [40,41,42,43], while, in this study, the growth of a*tcup1* lines and *AtCUP1*-edited lines were not affected by mutation of *AtCUP1* compared to WT. The possible reason is that OsNramp1, OsNramp5, OsHMA3 and OsHMA2 are also involved in the transportation of nutrient elements, including Fe^2+^, Zn^2+^ and Mn^2+^. The functional disruption of these transporters consequently interfered with nutrient absorption while reducing Cd uptake. Unlike these transporters, AtCUP1 was a major transporter of non-metal nutrient elements, and the loss of function mutation did not lead to a lack of nutrients in the plant.

Since *A. thaliana* is closely related to oilseed rape, and since they have high homology in both gene sequence and locus, orthologous genes in *A. thaliana* and *B. napus* played, in most cases, similar roles [44,45,46]. Therefore, *BnCUP1* was edited by the CRISPR/Cas9 system based on the editing strategy used in *AtCUP1* editing, in order to investigate whether Cd uptake and accumulation in the *B. napus* were affected. In the end, according to the hydroponic experiment and field experiment, it was found that *BnCUP1*-edited lines reduced the Cd accumulation by decreasing the Cd uptake in roots. In addition, Fe, Zn and Mn concentrations were detected in *BnCUP1*-edited lines, and it was found that the *BnCUP1*-edited lines maintained metal steadiness. Furthermore, *BnCUP1*-edited lines did not cause obvious defects in yield-related agronomic traits.

In conclusion, in this study, a new gene (*AtCUP1*) related to Cd accumulation was found by a Cd-sensitive yeast system, and its ability to absorb Cd was verified in *A. thaliana*. Finally, utilizing CRISPR/Cas9 technology, we created the *BnCUP1*-edited *B. napus* lines, aiming at the regions where it may potentially be applicable. It was further confirmed that the *BnCUP1* gene could balance low-Cd accumulation and crops yields well. The *BnCUP1*-edited lines greatly reduced the Cd concentration, with no negative effects on yield. This provides an important genetic resource for the cultivation of edible and fodder oilseed rape with a low risk of Cd.

## Figures and Tables

**Table 1:** Statistics of agronomic characters of three lines grown in Cd-contaminated soil.

Material	Branch Number	Plant Height (cm)	Silique Number per Plant	Number of Seeds per Silique	1000-Seed Weight (g)	Yield/Plant (g)
Westar	7.47 ± 0.47	138.38 ± 4.90	246.40 ± 16.09	13.40 ± 0.52	3.70 ± 0.10	12.07 ± 0.89
*S12*	8.80 ± 0.70	147.93 ± 3.38	299.67 ± 21.30	16.64 ± 0.48 **	3.43 ± 0.08	17.04 ± 1.27 **
*S34*	8.67 ± 0.77	150.65 ± 2.50 *	303.13 ± 26.30	17.59 ± 0.40 **	3.42 ± 0.10 *	17.68 ± 1.29 **

Note: The data represents the mean ± SD; statistical analysis was performed by Student’s *t-*test (* *p* < 0.05, ** *p* < 0.01).

## Data Availability

Not applicable.

## Supplementary Materials

The following supporting information can be downloaded at: https://www.mdpi.com/article/10.3390/cells11233888/s1, Figure S1: Phylogenetic tree of the MFS members in *Arabidopsis thaliana*. Figure S2: Prediction of subcellular localization and transmembrane domain of AtCUP1. Figure S3: Display of editing results of *AtCUP1*-editing lines (*CR-1*, *CR-2* and *CR-3*). Figure S4: Cadmium localization in the root and hypocotyl tissues of *CRISPR-AtCUP1* lines exposed to 0 or 60 µM Cd for 7 d. Figure S5: Alignment of *CUP1* homolog sequences identified from *A. thaliana* (*AtCUP1*) and *B. napus* (*BnCUP1-1a*, *BnCUP1-1b*, *BnCUP1-1c* and *BnCUP1-1d*). Figure S6: Relative expression levels of each copy of *BnCUP1*. Figure S7: Display of editing results of *BnCUP1* gene editing lines (*CRISPR-S12* and *CRISPR-S34*). Table S1: Primers used in the present study. Table S2: Detection of potential off-target sequences. Table S3: Detection of physical and chemical properties of the soil. Table S4: Statistics of agronomic characters of three lines grown in the control soil.
