# Supplementary material for: Editing of a Novel Cd Uptake-Related Gene CUP1 Contributes to Reducing Cd Accumulations in Arabidopsis thaliana and Brassica napus
Source: Cells. 2022 Dec 1;11(23):3888. doi: 10.3390/cells11233888 (PMC9739810; doi:10.3390/cells11233888)
Supplement: Supplementary file 1 [file cells-11-03888-s001.zip › cells-1993621-supplementary.pdf]

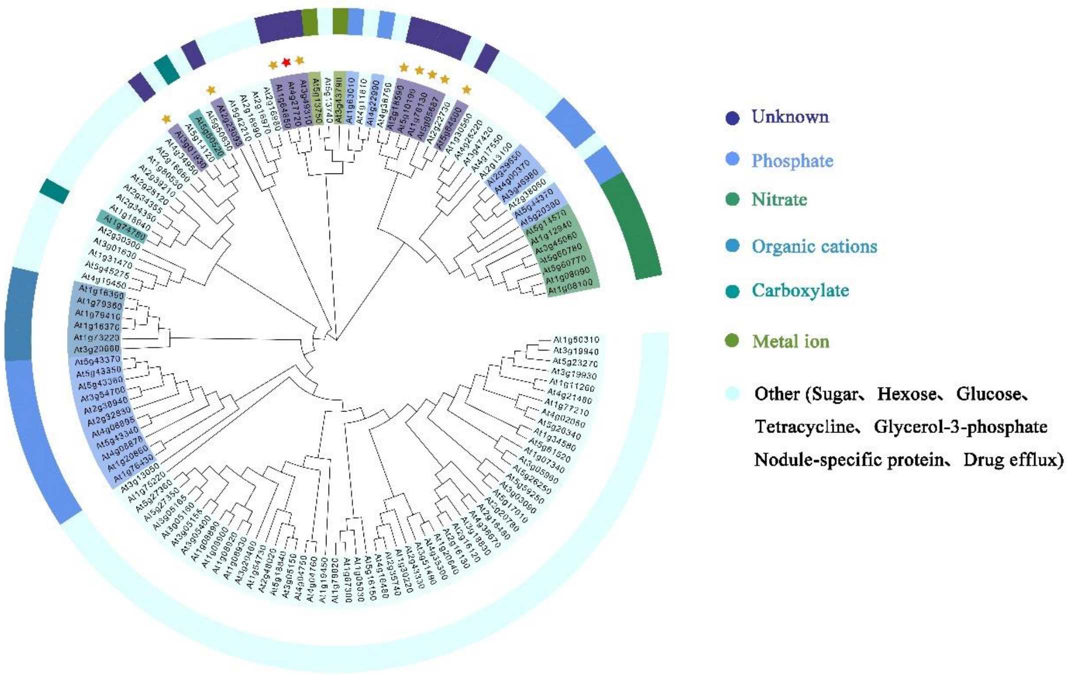

Figure S1 Phylogenetic tree of the MFS members in *Arabidopsis thaliana*.

MFS proteins with different transport substrates are represented with different colors. MFS proteins with unclassified substrates are represented with stars. AtCUP1 is marked with red stars. The gene symbol IDs of these unclassified genes are as follows: A: At1G64650; B: At1G78130; C: At3G49310; D: At4G27720; E: At5G10190; F: At5G65687; G: At2g23093; H: At3g01930; I: At5g64500; J: At2g18590.

(a)

Protein	Plasma membrane	Tonoplast	Endoplasmic reticulum membrane
AtCUP1	9	3	2

(b)

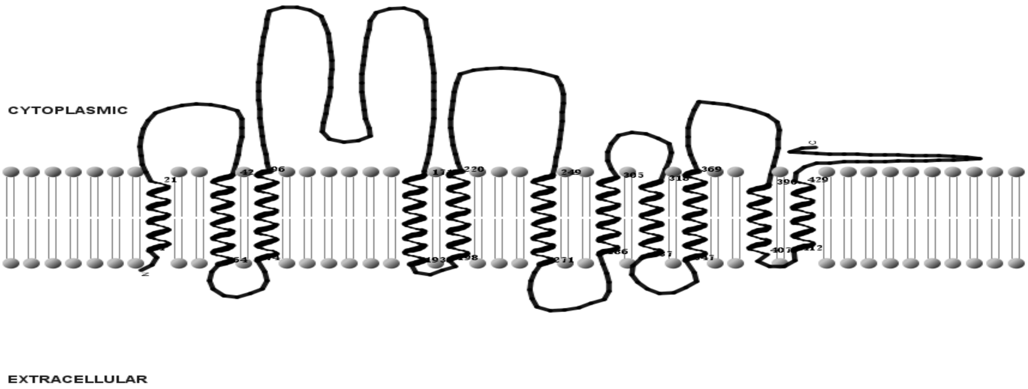

Figure S2 Prediction of subcellular localization and transmembrane domain of AtCUP1
(a) Subcellular localization of AtCUP1 protein, predicted by using WoLF PSORT (https://wolfpsort.hgc.jp) website. (b) Transmembrane domain of AtCUP1 protein, predicted by using TMHMM and showed by TMRPres2D.

SgRNA-reference GGAGCTTAGCAAGAACAACAAGG

CR-1 GTGAGTTTAGCAAGAACAACAAGG + T

SgRNA-reference GATTCCTCCACCTTTCACCTTGG

CR-2 GATTCCTCCACCTTTCAACCTTGG + A

SgRNA-reference GGTAAATGCATTCCCTATCACGG

CR-3 GGTAAATGCATTCCCTAATCACGG +A

Figure S3 Display of editing results of *AtCUP1*-editing lines (*CR-1*, *CR-2* and *CR-3*)
The genome sequence of the wild-type is represented by reference sequence in *A. thaliana.* The underlined part represents the sgRNA and the red part represents PAM. The sequences of *CR-1*, *CR-2* and CR-3 represent the gene edited sequences of *CRISPR-AtCUP1.*

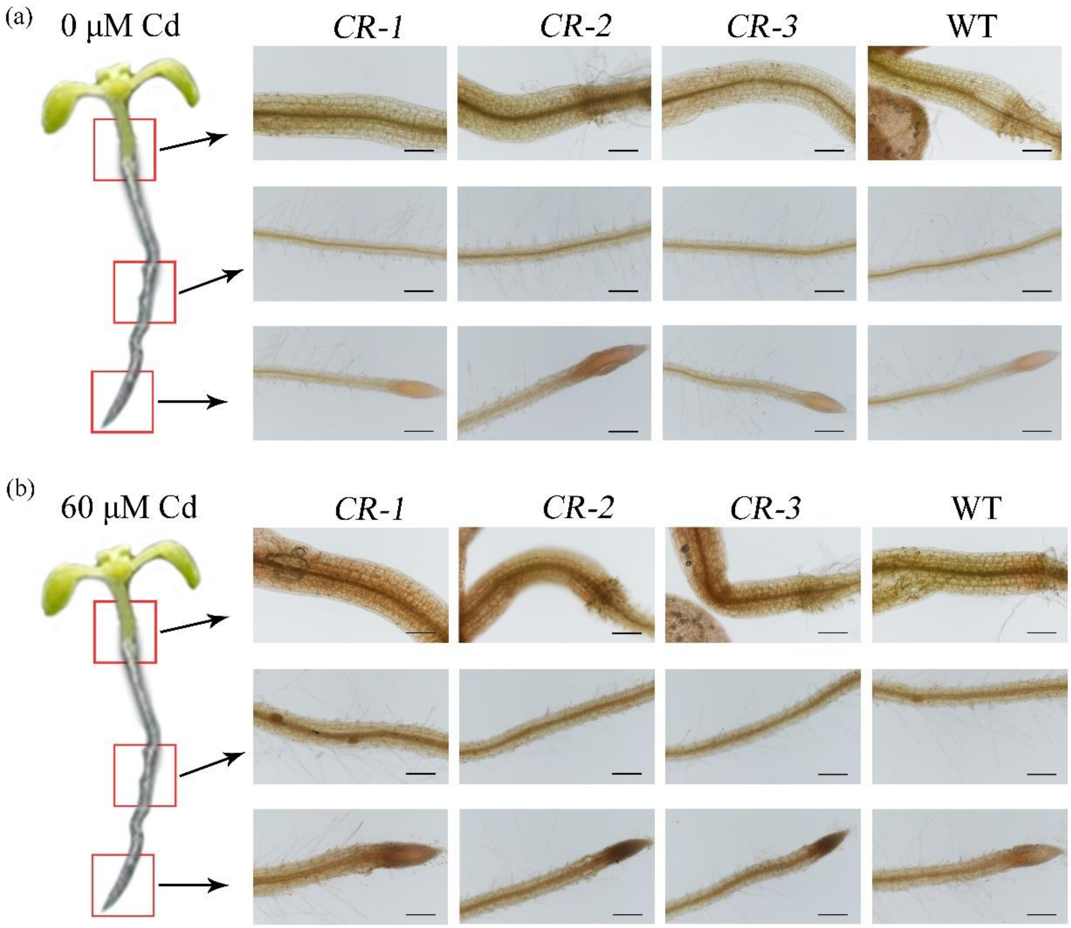

Figure S4 Cadmium localization in the root and hypocotyl tissues of *CRISPR-AtCUP1* lines exposed to 0 or 60 μM Cd for 7 d

Cadmium - dithizone precipitate was reddish-brown precipitate. Bars= 500 μm.

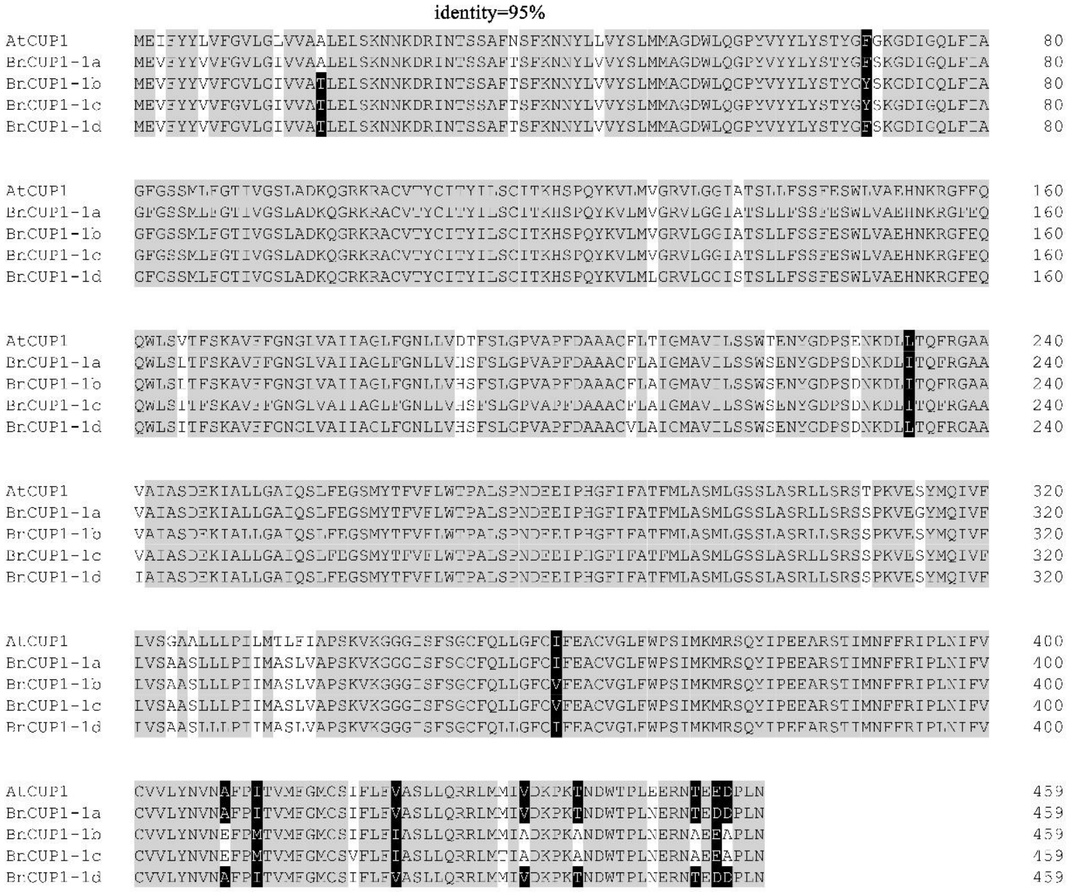

Figure S5 Alignment of CUP1 homolog sequences identified from *A. thaliana* (*AtCUP1*) and *B. napus* (*BnCUP1-1a*, *BnCUP1-1b*, *BnCUP1-1c* and *BnCUP1-1d*). The amino acid sequence differences are highlighted in White and black boxes.

a

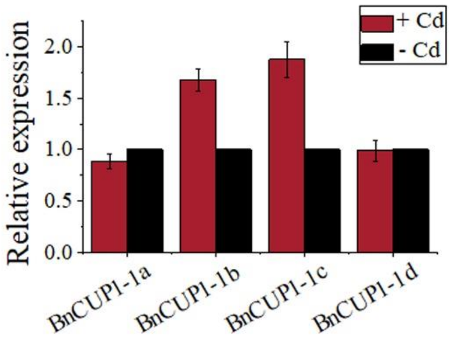

b

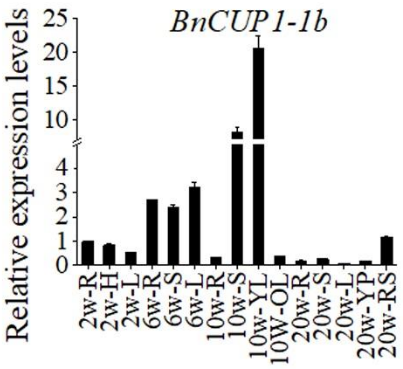

c

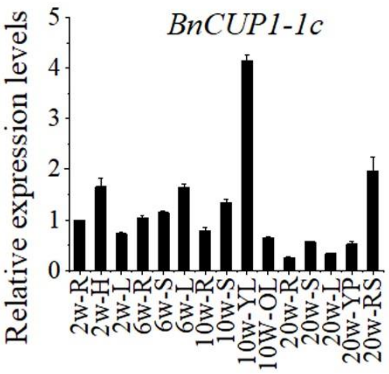

Figure S6 Relative expression levels of each copy of *BnCUP1*

(a) Relative expression levels of *BnCUP1* copies before and after Cd stress. The relative expression level after Cd treatment is shown in red, and the relative expression level without Cd treatment is shown in black. (b) The relative expression level of *BnCUP1* copies in different tissues at different stages of rapeseed development were detected via qPCR. 2W, 6W, 10W and 20W represent *B. napus* aged 2 weeks, 6 weeks, 10 weeks and 20 weeks, respectively. R indicates the root, H indicates the hypocotyl, L indicates the leaf, S indicates the stem, YL indicates the young leaf, OL indicates the old leaf, YP indicates the young silique, RS indicates the rape seed.

SgRNA-Reference		TTTGGTTCCTCTATGCT- CTTTGG
S12	*BnCUP1-1c*	TTTGGTTCCTCTATGCTACTTTGG
	BnCUP1-1b	TTTGGTTCCTCTATGCTTCTTTGG
SgRNA-Reference		GTAGCACCTTCCAAAGT -AAAGGG
S34	*BnCUP1-1c*	GTAGCACCTTCCAAAGTAAAAGGG
	BnCUP1-1b	GTAGCACCTTCCAAAGTAAAAGGG

Figure S7 Display of editing results of *BnCUP1* gene editing lines (*CRISPR-S12* and *CRISPR-S34*)

The genome sequence of the wild-type is represented by reference sequence in *B. napus.* The underlined part is PAM. The red font represents base changes in the edited lines.

Table S1 Primers used in the present study

role	primers	Sequences (5' to 3')
qPCR assay of Cd uptake candidate genes in Arabidopsis	At1G64650 -qpcr-F	TACTTCGTGGTGTTCGGTGG
	At1G64650 -qpcr-R	TCCCCAGCCATCATGAGAGA
	At1G78130-qpcr-F	GATTTGGTCCGGGAAGCTGA
	At1G78130-qpcr-R	CCCGTGTGAGAACCCAATGA
	At2g18590-qpcr-F	CGTCCCACAGTTTTTGCGTT
	At2g18590-qpcr-R	TGCATGTCCAACCCCATTGA
	At2g23093-qpcr-F	TGCGTGGAGGGATGATAAGC
	At2g23093-qpcr-R	TGTGGTGACTTTCCCCATCG
	At3g01930-qpcr-F	CTCTTCTCGTCGGCTCTGTC
	At3g01930-qpcr-R	TGAGGATACACATAGCCCATAAAGG
	At3G49310-qpcr-F	CCAGCTCTTAGCCCAAACGA
	At3G49310-qpcr-R	GAAGTGAAGCAGCCGAGACT
	At4G27720-qpcr-F	TTGGGGTTTTGGGTCTCGTC
	At4G27720-qpcr-R	AACCAATCACCCGCCATCAT
	At5G10190-qpcr-F	TAACCGAGCTCACGTCATCG
	At5G10190-qpcr-R	GACTAGGGACTGAATCGCGG
	At5g64500-qpcr-F	AGGGGTCACAGTTGTATGCG
	At5g64500-qpcr-R	ACCCTGTGTGGCAAAGACAA
	At5G65687-qpcr-F	GTCTCCATTGCGTGAGACCA
	At5G65687-qpcr-R	AGCTGCGAGGAAGAGGATTG
Yeast functional experiments	PYES2-A-F	CGGGGTACCATGGAGATCTTCTACTTCGTGGT
	PYES2-A-R	CCGGAATTCTTATGGGTTTAGAGGGTCAGC
	PYES2-B-F	CGGGGTACCATGAAGGCGGAGACGATGAC
	PYES2-B-R	CCGGAATTCTTATCTAGCTTGTAAATTGGGGT
	PYES2-C-F	CGGGGTACCATGGAGGTTTTCTACTACTTGG
	PYES2-C-R	CCGGAATTCTCAGAGGGTAAGAGGATCAACTT
	PYES2-D-F	CCCAAGCTTATGGAGATTTTCTACTACTTGGT
	PYES2-D-R	CCGGAATTCTCATATGTTGAGGGGATCTTCTT
	PYES2-E-F	CGGGGTACCATGAAGTCGGAGACTTTAACATT
	PYES2-E-R	CCGGAATTCTTAACTCTCATTCTGATGCTG
	PYES2-F-F	CGGGGTACCATGACGAGAGTTGGCCAGAGA
	PYES2-F-R	TGCTCTAGATTAGGCGAGAGTAGAGTTCTCT
	PYES2-G-F	CGGGGTACCATGGGTGTCGTTATTGAGACCT
	PYES2-G-R	CCGGAATTCTCATAATTTGTGCCAATCAT
	PYES2-H-F	CGGGGTACCATGGCGAGGACGACGCGAGAAAGAG
	PYES2-H-R	CCGGAATTCTCAGTTGCGGGTTTTGCCGTAGAGA
	PYES2-I-F	CGGGGTACCATGGATGTTGACGGAGAAGGTG
	PYES2-I-R	CCGGAATTCTCATGCTTCCTGGAGAAGAGG
Construction of *AtCUP1* overexpression vector	35s::AtCUP1-F	GCTCTAGAATGGAGATTTTCTACTACTTGGT
	35s::AtCUP1-R	CCCCCGGGTATGTTGAGGGGATCTTCTT
	SALK-0454-LP	ATGTAGCTGGATTGAGCATGG

Identification of *AtCUP1* mutants	SALK-0454-RP	CTTAGGAGCCACACGATCTTG
	LBb1.3-BP	ATTTTGCCGATTTCGGAAC
Subcellular localization	PBI221-AtCUP1-F	GCTCTAGAATGGAGATTTTCTACTACTTGGT
	PBI221-AtCUP1-R	GCGGATCCTATGTTGAGGGGATCTTCTT
Analysis of *AtCUP1* promoter activity	Pro-4G-F	CCCAAGCTTTAGTTCTGAATGGTGAGGAAGAGA
	Pro-4G-R	CGCGGATCCTGTCGTTCGATCCAGAAACTAT
CRISPR-*AtCUP1* vector construction	U6-26-T1-F	GATTGGTTTTGGGTCTCGTCGTCG
	U6-26-T1-R	AAACCGACGACGAGACCCAAAACC
	U6-29-T2-F	GATTGCTTGTCTATTCGCTCATGA
	U6-29-T2-R	AAACTCATGAGCGAATAGACAAGC
	U6-26-T3-F	GATTGATTCCTCCACCTTTCACCT
	U6-26-T3-R	AAACAGGTGAAAGGTGGAGGAATC
	U6-29-T4-F	GATTGCTCGAATATACAGAACCCA
	U6-29-T4-R	AAACTGGGTTCTGTATATTCGAGC
	U6-26-T5-F	GATTGGTAAATGCATTCCCTATCA
	U6-26-T5-R	AAACTGATAGGGAATGCATTTACC
	U6-29-T6-F	GATTGTGTTGCAAGTCTCCTACAA
	U6-29-T6-R	AAACTTGTAGGAGACTTGCAACAC
Identification of *AtCUP1*-edited lines	T1-T2edi-F	AGAAGAATTGAGAGATAACAGAGAG
	T1-T2edi-R	AGGAAGTAAAATCTAAAAGGGGCAA
	T3-T6edi-F	CTATTGGGTGCTATCCAGTCGCTCT
	T3-T6edi-R	CTTCTTCTGTGTTTCTTTCT
qPCR analysis of *BnCUP1* under Cd stress	BnCUP1-1a-qpcr-F	TCGTTGGATCTCTTGCCGAC
	BnCUP1-1a-qpcr-R	CAGCGACAAGCCATGATTCG
	BnCUP1-1b-qpcr-F	CCCTGAGGAAGCAAGAAGCA
	BnCUP1-1b-qpcr-R	GCCTTTGGCTTGTCAGCAAT
	BnCUP1-1c-qpcr-F	GCTCTAAGCCCCAACGATGA
	BnCUP1-1c-qpcr-R	AATAGTGAGGCCGCAGACAC
	BnCUP1-1d-qpcr-F	GCAATCGCCATAGCCTCTGA
	BnCUP1-1d-qpcr-R	CATCGTTGGGGCTTAGAGCA
CRISPR-*BnCUP1* vector construction	S12-s1-BsF	ATATATGGTCTCGATTGTTGGTTCCTCTATGCTCTTGTT
	S12-s1-F0	TGTTGGTTCCTCTATGCTCTTGTTTTAGAGCTAGAAATAGC
	S12-s2-R0	AACTGTATTGAGGAGAATGCTTCAATCTCTTAGTCGACTCTAC
	S12-s2-BsR	ATTATTGGTCTCGAAACTGTATTGAGGAGAATGCTTCAA
	S34-s3-BsF	ATATATGGTCTCGATTGTAGCACCTTCCAAAGTAAAGTT
	S34-s3-F0	TGTAGCACCTTCCAAAGTAAAGTTTTAGAGCTAGAAATAGC
	S34-s4-R0	AACAAACAGTCCTACACACGCCCAATCTCTTAGTCGACTCTAC
	S34-s4-BsR	ATTATTGGTCTCGAAACAAACAGTCCTACACACGCCCAA
Identification of *BnCUP1*-edited lines	S12-1a-s2-edi-F	TACTCTACTACTATGAGTTAGAGACTGGTTGT
	S12-1a-s2-edi-R	AATAAAAGCATCAGAAAGAGAGGAAACTCAC
	S12-1a-s1-edi-F	TATGTTCTTGTACTTAATGGCAAATCTCAACA
	S12-1a-s1-edi-R	ATAAACCTTTGGTAACTTTACCTTATTGTGCT
	S12-1b-s1-edi-F	GTTTATAGGAAGGTGGAGTTTTGGTATATGTC
	S12-1b-s1-edi-R	AACATAATAAAAGCATGAGAGAGAGAGAGAGA

	S12-1c-s1-edi-F	TATGGGTATAGCAAAGGGGACATTGG
	S12-1c-s1-edi-R	AATATGTAAGTAATACAGTACGTAACGCACGC
	S12-1d-s1-edi-F	ATGATCTAACCGGAGTTTGATATTTTGTGAAT
	S12-1d-s1-edi-R	TTGTTGAGATTTGCCATTTAGTAGTGTAAGAA
	S34-1a-edi-F	TGTGGGTTCTTCTTTGCGTGC
	S34-1a-edi-R	GATTGTGCTTCTGGCTTCCTCA
	S34-1b-edi-F	TGCTGCCTCACTATTGCTTCCA
	S34-1b-edi--R	GTGCTTCTTGCTTCCTCAGGG
	S34-1d-edi-F	CGCTTCGATGCTCGGCAG
	S34-1d-edi-R	TGGCTTCCTCAGGTATGTATTGGG
	S34-1c-edi-F	GCATCTCGTCTGTTGTCTCGC
	S34-1c-edi-R	TGGCTTCCTCAGGGATGTATTGG
	S12-C07-F	CCTCGACTTGATCCGACTTGT
	S12-C07-R	CTCGGCGATCCCTTTTACCT
	S12-C08-F	TACGTCCTCGTCTTCTCCGT
	S12-C08-R	TGCTCAGCAATGAGCCAAGA
	S12-CNN-F	CACGTCAGAATGTTGATTTCCTCA
	S12-CNN-R	TGCAACCTTGTACCTACACAA
	S12-C03-F	TCGCTTATCTTGCGCCTCAC
	S12-C03-R	CCGGAGAAACAGAGGCTGAG
Off-target detection of *BnCUP1*-edited lines	S12-A03-F	ACAAGCTCCTGTAACCGGC
	S12-A03-R	TTGCCGGAAAGTCAGAGGTC
	S34-A10-F	TGCGTGTCCTACTTGTGCTT
	S34-A10-R	GTCACAACATTTCCCAATGGCT
	S34-C03-F	TTGGTCCCTACAGGCCAAC
	S34-C03-R	TCCCATCCAAGGAACTGGCTA
	S34-C04-F	TGAGGAAGAACCAGCAGCAG
	S34-C04-R	CATCCAAGGAGCTAGCCAGG
	S34-C08-F	AGTGCCGACAGTGAAAACCA
	S34-C08-R	CCGAGCTTCTGACGCTACTA

Table S2 Detection of potential off-target sequences

Sequence name	Sequence	putative off-target locus	off-target (Y/N)
CRISPR-S12-sgRNA1	TTTGGTTCCTCTATGCTCTTTGG	-	-
OFF_1	ATTGGTCCCTCCATACTCTTCGG	BnaC07g43370D	N
OFF_2	TTCGGCTCCTCTATGCTCTTTGG	BnaC08g21010D	N
CRISPR-S12-sgRNA2	ATGGTGGGCCGTGTGCTGGGAGG	-	-
OFF_1	ATAGTGGCCCGTGTGCTTCGAGG	BnaCnng06240D	N
OFF_2	AAGGTGAGCCGTAAGCTGGGAAG	BnaC03g35330D	N
OFF_3	AGGGTGAGCCGTAAGCTGGGAAG	BnaA03g30060D	N
CRISPR-S34-sgRNA3	GTAGCACCTTCCAAAGTAAAGGG	-	-
OFF_1	GTAACACCTTCCAAAATATATGG	BnaA10g08980D	N
OFF_2	GTAGCACAAACCGAAGTAAACGG	BnaC03g22100D	N
OFF_3	GTAGCACAAACCGAAGTAAACGG	BnaC04g06140D	N
OFF_4	GGAGCACCTTCCACAATATATGG	BnaC08g36380D	N
CRISPR-S34-sgRNA4	AGGCGTGTGTAGGACTGTTTTGG	-	-
OFF_	None	-	-

Table S3 Detection of physical and chemical properties of the soil

Soil type	PH	Total N (g/kg)	Total P (g/kg)	Total K (g/kg)	Organic matter (g/kg)	Cd concentration (mg/kg)
soil 1	5.80±0.07	1.35±0.05	0.78±0.05	6.03±0.01	21.68±0.77	5.12±0.31
control	6.62±0.09	1.57±0.05	0.62±0.01	8.45±0.06	26.05±0.83	0.14±0.02

Note: Soil 1 is the experimental soil; control is the field soil with low-Cd concentration.

Table S4 Statistics of agronomic characters of three lines grown in the control soil

Material	Branch number	Plant height (cm)	silique number per plant	number of seeds per silique	1000-seed weight (g)	Yield/plant (g)
Westar	9.67±0.67	157.80±2.54	352.47±12.91	19.60±0.50	3.90±0.05	26.91±1.23
S12	9.53±0.48	156.53±3.14	348.27±11.84	19.92±0.49	3.72±0.08	25.62±0.82
S34	9.80±0.56	160.27±2.95	351.40±16.02	20.356±0.35	3.69±0.07*	26.22±1.12

Note: The data represent the mean ± SD; Significant differences were tested using the least significant difference (LSD) method (*$P < 0.05$, **$P < 0.01$).
